# Supplementary material for: Are carbon emissions trading and green financial instruments synergistic? -Comprehensive quantitative research based on content analysis
Source: PLoS One. 2024 Mar 7;19(3):e0298601. doi: 10.1371/journal.pone.0298601 (PMC10919601; doi:10.1371/journal.pone.0298601)
Supplement: S1 Data — (ZIP) [file pone.0298601.s001.zip › Data/地级市Z_F工作报告.zip!/░▓╗╒/┬φ░░╔╜╩╨/└┤╫╘╣1⁄2╓┌║┼í╛┬φ┐╦╩2╛▌═°í┐_2016.docx]

# **政府工作报告（2016）**

【字体大小：[大](http://zwgk.mas.gov.cn/openness/detail/content/javascript:doZoom(18)) [中](http://zwgk.mas.gov.cn/openness/detail/content/javascript:doZoom(15)) [小](http://zwgk.mas.gov.cn/openness/detail/content/javascript:doZoom(14))】

——2016年1月6日在马鞍山市第十五届人民代表大会第五次会议上

　　市长 魏尧

各位代表：

现在，我代表市人民政府，向大会报告政府工作，请予审议，并请市政协委员和其他列席人员提出意见。

一、2015年工作回顾和“十二五”发展成就

2015年是我市发展很不平凡的一年，面对经济总体下行和资源型城市转型的双重压力，我们在省委、省政府和市委的坚强领导下，在市人大、市政协的监督支持下，认真贯彻落实党的十八大、十八届三中、四中、五中全会和总书记系列重要讲话精神，牢牢把握稳中求进、进中求好、好中求快的工作总基调，主动适应经济发展新常态，全力推进转型升级、加快发展，较好地完成了市十五届人大四次会议确定的主要目标任务，全市经济稳中有进、稳中有好，社会大局和谐稳定。

预计，全市实现地区生产总值1360亿元，增长9.4%；财政收入210亿元，增长3.6%；固定资产投资1876亿元，增长12%；社会消费品零售总额419亿元，增长12.2%；城镇居民人均可支配收入35100元，增长7.8%；农村居民人均可支配收入16400元，增长9.6%。

（一）经济运行稳中有进。工业支撑作用明显，实现规上工业增加值600亿元，增长9.6%；新增规上工业企业189家，总数达1053家；工业用电量增长4.7%；新增工业项目供地5155亩，增长25.2%。招商引资成效显著，中兴皖通产业园、华谊名品产业园、科达洁能新材料等一批10亿元以上项目成功签约；5000万元以上工业项目新签约600个、新开工320个、竣工200个；完成新开工项目投资170亿元、续建项目投资260亿元、技改项目投资310亿元，均超额完成目标任务；预计实际利用省外亿元以上项目到位资金620亿元，外商直接投资19.4亿美元，均增长10%。落实“四督四保”要求，推动项目建设提质提效，仙乐健康产业园、世界村橡塑新材料、嘉寓智能门窗等208个项目开工建设，日发纺机、同心石化、威达环保等126个项目竣工投产。现代服务业加快发展，服务业占GDP比重达33.1%，提高1.2个百分点。“一基地四园区” 建设稳步推进，工业设计中心建成运行，软件园入驻企业突破200家。当涂县获批全省首批电子商务示范县，青年电商园入驻企业50家，饮马湖互联网创业城开园运营。举办第二届中国（马鞍山）互联网大会等各类展会77场。居然之家建成开业，东誉城招商中心基本建成，解放路等3条商业街获评省级特色商业示范街。文旅产业融合发展，采石古镇、凌家滩文化公园等重点项目稳步推进，采石风景区积极争创5A级旅游景区，褒禅山等2个景区获批4A级旅游景区，完成旅游总收入172亿元。房地产市场呈现“量增价稳”态势，市区商品房成交面积、金额分别增长31%、20%。十七冶集团晋升为“双特双甲”资质建筑企业。农业总量稳步增长，实现农业增加值增长4.5%，规上农产品加工产值增长18%。规模经营比重进一步提高，全市土地流转面积102万亩，流转率达55.1%。农业新型经营主体总量迅速壮大，新增市级农业产业化龙头企业18家、农民专业合作社123家、家庭农场104家。当涂县被评为国家现代农业示范区，马鞍山国家农业科技园区获批组建。

（二）创新转型取得实效。“三项培育”工作成效明显，新培育科技小巨人企业50家；引进高层次人才团队9家；建诚国际在澳大利亚主板上市，华骐环保已上报证监会申请上市，经纶文化在省证监局辅导备案，圆融光电、普邦担保等10家企业在“新三板”挂牌，22家企业在省股权交易中心挂牌。全市银行业金融机构贷款余额1174亿元，社会融资总规模456亿元，其中直接融资147亿元。深入持久服务企业，完善市与马钢常态化对接机制，推进钢城融合发展；出台《鼓励和引导企业兼并重组的意见》，银阳光伏、东海机床等28家企业实施兼并重组，涉及金额30亿元；兑现产业扶持政策资金8亿元，惠及企业1300余家；设立1亿元的创投基金，采取“先债权后股权”方式对16家优质中小企业给予支持。支持企业开展创新，为396家企业发放“科技创新券”8300多万元；设立6000万元的创业天使投资引导基金，建成各类“双创”平台4家；新增院士工作站1家、博士后工作站8家；新认定高新技术企业56家、高新技术产品262个，全社会研发投入占GDP比重达2.5%。打响调结构转方式促升级攻坚战，启动建设首批8个战略性新兴产业集聚发展基地，重点发展轨道交通装备、高端数控机床、现代化工和绿色食品产业，培育发展电子信息、节能环保、生物医药和高端装备制造及机器人产业，其中轨道交通装备产业基地入选全省首批战略性新兴产业集聚发展基地，实现战略性新兴产业产值增长14.5%。获批各类专项资金和债券30.4亿元，争取发行企业债券25亿元。获批国家信息消费示范城市，信福小屋等2个项目被评为全国信息消费创新应用示范项目。全国质量强市示范城市创建工作扎实推进，国家机床产品质量监督检验中心主体竣工。当涂经开区获批省级知识产权示范园区，中冶华天、泰尔重工等5家企业荣获“国家级知识产权优势企业”称号。授权发明专利775项。新创中国驰名商标5件。

（三）改革开放活力增强。完成新一轮市、县（区）政府职能转变和机构改革，全面建立市县乡三级政府权力清单、责任清单并实行动态管理。推进商事制度改革，实施“三证合一”、“一照一码”，新登记注册企业5559家。深化农村改革，在全省率先开展“整市推进”农村土地确权登记颁证、农村集体资产股份制改革，建成区域性农村产权交易中心。深入推进医药卫生体制改革，合理调整医疗服务价格，推进分级诊疗，全面破除“以药补医”机制。稳妥推进户籍制度改革，实行一元化户口登记制度。深入推进以港兴市战略，长江马鞍山段航道整治工程开工建设，慈湖综合码头、天顺港集装箱堆场建成，长江港口货物仓储加工基地加快建设，郑蒲港铁路项目稳步推进，完成港口吞吐量9100万吨，增长12%，集装箱18万标箱，增长71%，其中郑蒲港集装箱量超1万标箱。综合保税区申建扎实推进，马鞍山口岸郑蒲港对外扩大开放获得国务院批准，进口肉类指定口岸成功获批，进口粮食指定口岸获准筹建。实现外贸进出口29亿美元，完成外经营业额3.8亿美元。外事侨务和对台工作取得新成绩，成功举办第四届海峡两岸（马鞍山）电子信息博览会、第二届海峡两岸节能环保产业论坛，国台办批复支持我市设立“海峡两岸产业转型转移示范基地”。全面融入长三角一体化发展，成功承办长三角城市经济协调会第15次市长联席会议、2015年长三角合作与发展联席会议。对外交通建设加快，商合杭高铁含山段开工建设，马滁高速建成通车；205国道北段改建工程通车，347国道郑蒲港段完成改造、和县段加快推进；226省道二期顺利推进，367省道改建工程开工。

（四）生态环境品质提升。开展城市总体规划修改，加强控规编制和动态维护，推进各类专项规划编制，城市设计水平进一步提升。城建路建成通车，天宝路、冯桥路一期及宁安高铁站前广场等工程竣工，慈湖河路改造加快推进，综合客运中心枢纽站开工建设。新增城市绿道61公里、绿化面积473万平方米，新建污水处理配套管网60公里。新增各类保障房1.26万套、竣工8000套。超限超载治理成效明显，城市主干道全部实现机械化清扫。美好乡村建设持续推进，建成29个省级中心村，改造县乡公路78公里、乡村公路180公里、农村危桥48座、农村危房3760户，建成农村污水处理设施15座。慈湖河中游综合整治、滁河防洪治理工程基本完工，水阳江下游、裕溪河防洪治理工程顺利推进。科学有效应对滁河流域超历史最高水位险情，确保人民群众生命财产安全。扎实开展城镇“三治三增三提升”行动，建立全市“三线三边”、城市管理及美好乡村环境综合治理工作考核机制。抓好生态市创建，重点生态项目建设有序推进，当涂县获得省级生态县命名，含山县、博望区通过省级生态县（区）验收，5个乡镇创建为省级生态乡镇，12个村创建为省级生态村。强力推进突出环境问题专项整治，整治各类环境问题327项，依法查处各类环境违法行为83起，停产整治358家环境问题企业，关停取缔202家环境违法企业。全年PM10平均浓度87微克/立方米，比上年下降19.4%。水生态文明城市试点工作扎实推进。生活垃圾焚烧发电项目开工建设。完成土地整治24万亩、土地征收1.4万亩，获批建设用地1.6万亩、供地2万亩。标准化厂房建成198万平方米，在建43万平方米。万元GDP能耗下降7.1%，主要污染物排放总量控制在省下达指标内。

（五）人民生活持续改善。全年民生支出168.6亿元，占财政总支出83.4%，完成33项民生工程和10件民生实事年度任务。大力促进就业创业，新增城镇就业5.1万人，期末城镇登记失业率2.9%。建立机关事业单位养老保险制度，五项社保新增参保6.7万人。教育信息化三年行动计划圆满收官，在全省率先实现县区义务教育基本均衡全覆盖，新改扩建公办幼儿园16所，秀山实验小学、河海大学文天学院郑蒲港校区建成投入使用，开展职业教育五年一贯制办学改革，马鞍山师专与软件园共建软件和互联网学院。秀山医院建设积极推进，妇幼保健院门诊楼主体封顶，市精神病医院开诊运行。全国爱国卫生工作会议暨城乡环境卫生整治行动现场会在我市召开。计划生育工作全面加强。扎实推进养老服务业综合改革试点工作，新增养老院19个、床位1980张。积极开展国家公共文化服务标准化试点工作，基本建成市县乡村四级公共文化服务圈，节俭办好李白诗歌节等群众性文化活动。更加注重长效管理，推进文明创建工作常态化，顺利通过全国文明城市年度测评。全省首家众筹公益素食馆正式营业。广泛开展群众性体育活动，市第十一届运动会取得圆满成功。新闻出版、广播电视、文学艺术、社会科学、档案和地方志工作水平不断提高。妇女儿童、残疾人和慈善事业得到加强，红十字工作蓬勃开展，防灾减灾和气象现代化工作扎实推进。

深入开展法治宣传教育，市法治宣传教育中心建成开放。社会服务管理信息化平台试运行，花山区在全省率先开通“96345”便民服务中心。扎实推进信访制度改革，市县乡三级实现信访事项网上办理，开展信访积案化解专项活动，信访形势平稳向好。加强“平安马鞍山”建设，网格化巡防体系不断完善，社会大局保持稳定。深化安全生产责任体系、隐患排查治理体系、应急救援保障体系建设，有效防范各类生产安全事故发生。严格食品药品安全监管，保障人民群众饮食用药安全。民族团结、宗教和睦局面进一步巩固。国防教育、国防动员、人民武装、人民防空工作得到加强。驻马部队和广大民兵预备役人员在促进社会稳定、支持地方建设等方面作出了重要贡献。

过去的一年，我们扎实推进法治政府建设，主动向市人大及其常委会报告工作，向市政协通报情况，办理市人大议案9件、建议108件，市政协提案263件，办理质量不断提高。落实市政府部门与各民主党派、工商联和无党派人士对口联系制度，进一步加强协商民主建设。严格规范政府系统重大事项决策行为，完成重大事项合法性审查81项。深化政务公开，强化行政监察，推进审计监督全覆盖。实行县以下机关公务员职务与职级并行和乡镇工作补贴制度，扎实开展“四清一建”工作，深入推进村居干部职业化，全面完成社区“两委”换届任务。稳步推进公务用车改革，规范公务接待，巩固办公用房清理成果。全面推行效能点评制度，深入开展“走亲戚”活动。在全国城市“12345”公共服务热线第三方评估中，我市位列全省第一、全国第四。全面落实“三聚焦三查找三确保”要求，扎实开展“三严三实”专题教育，严格执行作风建设“两清单一办法”，加大廉政建设和反腐败力度，政府执行力和公信力进一步提升。

2015年全市经济社会发展取得的积极成效，为全面完成经市人大常委会批准调整后的“十二五”规划目标画上了圆满句号。

这五年，是迎难而上、实力增强的五年。地区生产总值跃上千亿元台阶，年均增长10.8%，人均生产总值达9600美元。累计实现财政收入突破千亿元，年均增长6.6%。累计完成固定资产投资超7000亿元，年均增长21%。累计完成社会消费品零售总额1550亿元，年均增长16.9%。如期完成全面小康社会计划目标，2013年、2014年连续两年位居全省综合目标管理绩效考核第3名。

这五年，是聚焦转型、结构优化的五年。规上工业企业数突破1000家，比五年前增长98%，规上工业增加值年均增长12.8%。战略性新兴产业总产值占规上工业总产值比重较2011年提升4.7个百分点。高新技术企业数达256家，比五年前增长2.5倍；高新技术产业产值是五年前的1.8倍。服务业总量扩大，新兴业态不断涌现，文旅产业融合发展，服务业占GDP比重年均提高约1个百分点。确立现代都市农业发展定位，农业增加值年均增长4.2%。

这五年，是拓展空间、夯实平台的五年。含山县、和县划入马鞍山，新成立花山区、博望区，市域面积和主城区面积分别扩大1.4倍和1倍，两次行政区划调整成为我市发展史上的里程碑，开启了一江两岸拥江发展新时代。长江大桥建成通车，宁安高铁投入运营，打通了跨江发展、融入长三角的快捷通道。慈湖高新区晋升国家级高新区，示范园区、郑蒲港新区先后成立并快速发展，多点支撑、多区联动格局不断夯实并逐步发力。

这五年，是深化改革、扩大开放的五年。投融资、工程建设、招标采购等六项管理体制改革不断完善，行政管理和农村综合改革扎实推进，教育、卫生、文化体制改革稳步实施。确立以港兴市战略，郑蒲港一期开埠运行，改变了安徽江北地区没有万吨级深水良港的历史。成功举办第四届全球外包大会。累计实际利用省外亿元以上项目到位资金2400亿元，外商直接投资75亿美元，对外开放水平不断提高。

这五年，是民生改善、社会和谐的五年。城镇和农村居民人均可支配收入分别跃上3万元和1万元台阶，年均分别增长11.5%和13.5%。累计投入资金167亿元，实施73项民生工程和民生实事。教育、卫生、社保等公共服务水平不断提高。蝉联全国文明城市称号，顺利通过国家卫生城市、全国双拥模范城市验收，成功创建国家公共文化服务体系示范区、全国和谐社区建设示范城市，城市知名度和影响力进一步提升。

各位代表，“十二五”以来我市取得这样的成绩实属不易、令人鼓舞。这是按照中央和省委、省政府的决策部署，在市委的坚强领导下，在市人大、市政协和社会各界的大力支持下，全市人民共同奋斗的结果。在此，我代表市人民政府，向辛勤工作在全市各条战线上的广大干部群众，向市人大代表、市政协委员、离退休老同志，向市各民主党派、工商联、无党派人士、人民团体和各界人士，向中央和省驻马单位、人民解放军、武警官兵和公安干警，向所有关心、支持、参与马鞍山建设发展的境内外投资者、海内外朋友们，表示衷心的感谢！

“十二五”以来政府工作行之有效的思路和方法，要在今后实践中继续坚持、不断完善。同时我们也清醒认识到，前进道路上还面临不少矛盾和困难，政府工作中还存在不少差距和不足，主要是：经济增长压力持续加大，去年的财政收入、战略性新兴产业产值、外贸进出口等指标完成情况与年初预期尚有差距；经济体量不大、结构不优仍是我们的主要矛盾，调结构转方式促升级任务艰巨，传统增长动力不足，新兴产业支撑不够，引进大项目、培育新产业尚未取得根本性突破；城乡资源承载压力日益加重，生态环境质量有待进一步改善；城乡区域发展不平衡，教育、卫生、养老等民生保障工作还需加强，政府职能转变有待进一步深化。对此，我们一定高度重视，采取有力措施，认真加以解决。

二、“十三五”发展的指导思想和目标任务

“十三五”时期是我市推动转型升级、加快发展极为重要的战略机遇期。随着国家“一带一路”、长江经济带战略深入实施，与皖江承接产业转移示范区等政策机遇同频共振，全省全面创新改革试验区建设和“调转促4105行动计划”向纵深推进，我市的区位交通优势将进一步凸显，转型升级方向将进一步明确，要素集聚能力将进一步提升。特别是近年来我们大桥高铁相继通车，多点支撑格局形成，园区建设加快推进，一批项目签约落地；干部群众齐心协力，干事创业氛围浓厚，发展思路更加清晰、精力更加聚焦、基础更加牢固，马鞍山迎来了厚积薄发的发展机遇期。同时也要看到，经济下行压力仍在持续，自身转型任务艰巨繁重，我们要时刻保持清醒头脑，科学把握规律，积极应对挑战，不断开创转型升级、加快发展新局面。

根据市委《关于制定国民经济和社会发展第十三个五年规划的建议》，“十三五”时期我市经济社会发展的指导思想是：高举中国特色社会主义伟大旗帜，全面贯彻党的十八大和十八届三中、四中、五中全会以及省委九届十四次全会、市委八届十六次全会精神，以马克思列宁主义、毛泽东思想、邓小平理论、“三个代表”重要思想、科学发展观为指导，深入贯彻总书记系列重要讲话精神，按照“五位一体”总体布局和“四个全面”战略布局，坚持发展是第一要务，坚持创新、协调、绿色、开放、共享发展，坚持转型升级、加快发展工作主题不动摇，以提高经济发展质量和效益为中心，以发展先进制造业为立市之基，以增进人民福祉、促进人的全面发展为出发点和落脚点，加快形成引领经济发展新常态的体制机制和发展方式，努力打造工业强市、生态强市、文化强市，全面建成更高水平小康社会，为基本实现现代化奠定更加坚实的基础。

在市委《建议》的指导下，市政府编制了“十三五”规划《纲要》。综合考虑，今后五年我市经济社会发展的总体目标是：全省争三强、人均居前列，全面建成更高水平小康社会。地区生产总值年均增长9%左右，突破2000亿元，向2400亿元冲刺；财政收入与经济同步增长；规上工业企业数突破2000家，规上工业增加值达到1000亿元、力争突破1200亿元；固定资产投资五年累计突破1万亿元。

重点抓好以下五个方面：

（一）坚持创新发展，开创转型升级新局面。把创新摆在发展全局的核心位置，加快建设创新型城市，着力构筑区域创新高地。集聚发展战略性新兴产业，力争到2020年，全市战略性新兴产业产值突破1000亿元。推动传统产业改造升级，加快发展现代服务业，提升现代都市农业，培育新产业新业态，促进文旅产业融合发展，打造优质高效现代产业新体系。抓好重点领域和关键环节改革，使市场在资源配置中发挥决定性作用，加快构建有利于转型升级、加快发展的体制机制。

（二）坚持协调发展，形成统筹推进新格局。坚持“组团式、网格化、田园城市”发展思路，提升主城区功能品质，增强城市组团内部功能，打通组团间快速通道，规划建设新的过江通道，推动一江两岸协调发展。推进以人为核心的新型城镇化，力争到2020年，常住人口城镇化率达70%。扎实开展美好乡村建设和“三线三边”环境整治，努力打造农民幸福生活美好家园。持续加强精神文明建设。推动军民深度融合发展。

（三）坚持绿色发展，彰显宜业宜居新优势。以创建省级和国家级生态文明建设示范区为抓手，巩固提升国家环保模范城市创建成果，认真落实主体功能区和工业集中区布点规划，促进人与自然和谐发展。推动低碳循环发展，积极发展新能源产业。加大环境治理力度，深入实施大气、水、土壤污染防治行动计划，推进工业污染源全面达标排放，实现城镇生活污水垃圾处理设施全覆盖，加大农业面源污染防治力度。推动资源节约高效利用，确保完成省政府下达的节能减排目标任务。

（四）坚持开放发展，构筑皖江开放新高地。深入推进以港兴市，加快郑蒲港新区建设，培育壮大临港产业，推进安徽江海联运枢纽中心建设，努力把我市打造成为外向型临港制造业基地和国家级物流进出口基地。坚持进口与出口并重、引进来和走出去并重、引资和引技引智并重，加强各类口岸建设，壮大外贸经营主体，打造全面开放新格局。坚持东向发展，深化长三角区域合作，加强对台合作交流。加快推进园区升级，切实提高招商实效，全面提升开放型经济发展水平。

（五）坚持共享发展，实现人民福祉新提升。坚持量力而行，尽力而为，办好民生工程和民生实事，提高公共服务共建能力和共享水平。坚决打赢脱贫攻坚战，力争在全省率先完成脱贫任务。大力发展教育事业，促进义务教育均衡发展，全面普及高中教育，加快现代职业教育体系建设。完善就业服务和社会保障体系，推动大众创业、万众创新。深化医药卫生体制综合改革，提高医疗服务水平，促进人口均衡发展，开展应对人口老龄化行动，推进健康城市建设。创新社会治理方式，加强信访调解、安全应急、平安建设和食品药品监管等重点工作，切实增强人民群众的幸福感和满意度。

三、2016年工作安排

今年是“十三五”发展的开局之年，也是推进结构性改革、加快“调转促”步伐的攻坚之年。我们要认真贯彻落实中央、全省经济和城市工作会议以及市委八届十六次全会精神，适应经济发展新常态，坚持改革开放，坚持稳中求进、进中求好、好中求快的工作总基调，坚持稳增长、调结构、惠民生、防风险，着力加强结构性改革，在适度扩大总需求的同时，去产能、去库存、去杠杆、降成本、补短板，增强持续增长动力，保持经济社会稳定健康发展，努力实现“十三五”发展良好开局，以优异的成绩向建市60周年献礼。

综合考虑，今年全市经济社会发展的主要预期目标为：地区生产总值增长9%，财政收入增长6%，固定资产投资增长11%，社会消费品零售总额增长11%，城镇居民人均可支配收入增长8%，农村居民人均可支配收入增长9%，城镇登记失业率控制在4%以内，单位GDP能耗、主要污染物排放总量控制在省下达指标以内。

为实现上述目标，我们将重点做好七个方面的工作：

（一）持续推动产业转型升级。把创新作为引领发展的第一动力，深入实施创新驱动发展战略，改造提升传统产业，加快培育新兴产业，着力打造长三角地区重要的先进制造业基地。

****壮大先进制造业实力。****深入推进工业经济倍增行动计划，实施利洁时桂龙药业、正崴科技园、诚富全降解材料等20个重点工业项目，新增规上工业企业180家，规上工业增加值增长10%。聚焦工业项目招商，力争5000万元以上工业项目新签约600个、新开工350个、竣工220个，新开工项目完成投资180亿元，续建项目完成投资270亿元。推动钢铁、汽车等传统产业智能化、绿色化改造，促进以互联网为代表的信息技术向设计、生产、市场等环节渗透，大力发展智能制造，完成技改投资350亿元。坚持钢城融合发展，加快马钢奥瑟亚现代化工、马钢埃斯科特钢等项目建设，支持马钢稳定经营、深化改革、创新转型。按照企业主体、政府推动、市场主导、依法处置的方法，积极稳妥化解产能过剩。支持华菱星马开展重组工作。更加重视支持中小企业发展，广泛宣传并兑现落实中央和省市出台的支持企业兼并重组、帮助企业降低成本以及金融支持实体经济发展等系列政策，搭建检验检测、要素交易、职业培训等公共服务平台，开展银政企、产学研、产销等对接活动，重视企业家队伍建设，支持企业开展创新创业，帮助企业解决实际困难，在全市上下营造尊重企业家、支持企业发展的浓厚氛围。增强企业质量、品牌和商标意识，培育一批知名品牌和驰名商标，积极争创全国质量强市示范城市、全国知名品牌示范区。

****加快战略性新兴产业发展。****高水平推进八大战略性新兴产业集聚发展基地建设，强化重大项目支撑，依托龙头企业带动，发挥产业基金引导作用，促进产业集聚化、高端化发展，力争战略性新兴产业产值增长11%。加快市经开区马钢城市轨道交通产业园和产学研基地建设，发展车轮、轮轴等轨道交通走行系统产业链。推进裕祥高端数控机床、恒永利高精度刀片项目建设，建成国家机床产品质量监督检验中心，提升博望区机床和刃模具产业智能化水平。加快瑞和工程新材料、名仕达新材料项目建设，新建和县精细化工基地公用码头，提高化工园区安全、绿色、循环发展水平。推进娃哈哈健康饮品、御香苑冷链保税物流园等项目建设，注重发挥蒙牛、达利等龙头企业支撑作用，打造绿色食品供应中心。加快瑞声科技、同兴环保、瑞邦生物、紫江包装等一批科技含量高、市场前景好、发展潜力大的优质项目建设，推进电子信息、节能环保、生物医药、高端装备制造及机器人等新兴产业发展。

****增强企业自主创新能力。****强化企业创新的主体地位和主导作用，支持重点企业牵头组建产业研究院和技术创新联盟，积极与国内外大院名校共建创新载体，加快科技成果产业化、资本化。抓好“三项培育”工作，新培育科技小巨人企业50家，力争培育上市（挂牌）企业20家、引进高层次人才团队20家。成立产业升级基金20支。高新技术产业产值占规上工业总产值比重达31%。落实好一系列支持创新创业的具体政策措施，培育一批创业示范基地、众创空间和专业孵化器，发挥天使基金、政策担保、财政补贴支持作用，继续在就医、上学、购房等方面给予最大便利，大力吸引企业高管、技术人才、高校教师和大学毕业生来马创新创业，在全社会营造尊重人才、支持创业的浓厚氛围。

（二）持续培育新兴服务产业。把握制造业和服务业融合发展新趋势，顺应个性化、多样化消费新潮流，加快培育新的消费增长点，推动现代服务业发展加快、比重提高、结构优化。

****大力发展生产性服务业。****鼓励有条件的制造业企业推进主辅分离，由生产型向生产服务型转变。扎实推进“一基地四园区”建设，实施盛通合运物流园、郑蒲港总部基地等10个重点服务业项目。支持工业设计中心发展，着力推动工业设计成果产业化。以云计算、大数据产业为突破口，加快东华村镇银行云平台、华拓金融服务外包基地、皖江信息产业园等项目建设，推动互联网与产业深度融合。重视发展现代金融产业，推进皖江金融中心项目建设，进一步健全金融服务体系，发挥金融支持实体经济发展的关键作用，着力防范化解金融风险。

****培育发展生活性服务业。****认真落实商业网点规划，推进东誉城、金鹰国际等项目建设，提升发展特色商业街区，加快社区商业服务网点、农村商品流通体系和农村“新网工程”建设，促进人气集聚和业态繁荣。推进国家信息消费示范城市建设，实施信息惠民示范工程，努力打造智慧城市。支持青年电商园、饮马湖互联网创业城建设，推动电子商务和快递物流业发展。建立购租并举的住房制度，发展住房租赁市场；更好发挥住房公积金作用，加大对进城农民购房贷款支持力度；提高棚改货币化安置率，促进房地产市场平稳健康发展。拓展服务、绿色、时尚、品质、农村等领域消费增长空间，促进消费扩大和升级。

****加快发展文教卫体产业。****积极对接皖南国际文化旅游示范区建设，深入推进文化旅游发展行动计划。突出运动健康休闲主题，加快凌家滩文化公园、濮塘休闲旅游度假区等10个重点文旅项目建设，完成采石风景区5A级旅游景区景观质量评定，力争大青山李白文化旅游区和鸡笼山-半月湖景区创建为4A级旅游景区，启动褒禅山香泉旅游度假区创建国家级旅游度假区工作，谋划大公圩水乡生态旅游区建设。抢抓高铁通车机遇，进一步整合区域资源、完善配套设施、提升景区品质、设计精品线路，努力打造国内旅游目的地城市。支持文旅集团做大做强，推动文旅产业发展壮大。发展创意设计、数字传媒等新型文化业态，支持经伦文化等企业发展，推进“创客+”文化创意园、玉产业园等项目建设。依托我市现有职教、幼教资源，积极发展教育产业，鼓励发展教育培训和互联网教育。完善居家养老服务体系，探索医养结合模式，大力发展养老产业。完善体育综合体运营管理，重点发展健身休闲、竞赛表演、场馆服务等体育服务业。积极承办举办各类重要会展、节庆活动和全国、全省性体育赛事，通过市场机制运作，扩大城市影响力和知名度，让全市人民生活更快乐。

（三）持续提高对外开放水平。充分发挥我市处于“一带一路”和长江经济带建设重要节点的优势，立足皖江，东向发展，强化合作，全面提升开放型经济水平。

****推进以港兴市。****充分利用好我市港口、岸线资源，以港口集团为主体，以资本为纽带，整合港口企业，打造新的马鞍山港口集团，并积极寻求对外合作，提升集聚辐射带动能力。全力支持郑蒲港新区发展，加快郑蒲港铁路建设，推进郑蒲港二期项目申报。加强与合肥港、舟山港、盐田港等港口合作，积极引进知名船代、货代、航运物流企业，吸引更多货物从我市集并中转，力争完成港口吞吐量1亿吨、集装箱20万标箱。实施长江马鞍山段航道整治及姑溪河、驷马山干渠航道疏浚工程，提升通航能力。加强开放平台建设，力争综合保税区尽快获批，郑蒲港口岸扩大开放通过验收，进口肉类指定口岸开埠运营，进口粮食指定口岸开工建设，大力发展现代临港经济，培育壮大装备制造、电子信息、食品加工、临港物流等主导产业，构建“口岸+产业+市场”一体化经济区。

****扩大对外开放。****加快对外贸易优化升级，重点支持高附加值和自主品牌产品出口，引导企业扩大先进技术装备、关键零部件和重要能源资源进口。深化关检“三个一”合作，推进企业就地报关直运工作，落实“三互”改革试点，加快大通关建设，提高贸易便利化水平。鼓励重点龙头企业扩大对外投资、开展国际并购，引导有实力的中小企业抱团出海、开拓海外市场。加强与台资企业交流合作，积极承接台资产业转移，充分发挥好“海峡两岸产业转型转移示范基地”的品牌效应。主动对接上海自贸区，积极参与长三角一体化发展，加强与南京在基础设施、公共服务、产业发展等领域合作，密切与合肥、芜湖等周边城市合作交流。

****加快园区转型。****坚持创新集聚、绿色集约、产城融合、示范带动的原则，创新园区发展理念和运营模式，聚焦园区首位产业和主导产业，不断做大体量和提升质量，推动各类开发园区错位发展、特色发展、集聚发展。始终把招商引资作为园区发展的头等大事，强化以商招商、产业链招商、专业化招商和领导带头招商，加强招商研究统筹工作，探索运用互联网思维开展招商活动，确保实际利用省外亿元以上项目到位资金690亿元，增长11%；实际利用外商直接投资21亿美元，增长9%。严把项目准入、投资强度和环境评价关口，提升开发园区资源集约利用水平。统筹规划建设开发园区公共服务设施，优化园区产业、城市、生态等功能布局。深入开展园区合作共建，推动宿州马鞍山现代产业园发展。支持当涂经开区创建国家级经开区、示范园区创建国家产城融合示范区。

（四）持续统筹城乡协调发展。加快推动一江两岸、城乡区域协调发展，在协调发展中拓展发展空间，在加强薄弱领域中增强发展后劲。

****构建综合立体交通体系。****坚持东向发展，与南京深度融合，加快推进轨道交通1号线、宁和城际项目前期工作，积极推进马濮旅游大道与苏340省道对接、314省道与苏341省道对接项目，加快367省道建设，力争247省道开工。连接一江两岸，完善对外通道，积极推进商合杭高铁含山段建设，做好巢湖-含山-和县-马鞍山城际铁路、九华路过江隧道等重大项目前期工作，加快347国道和县段、226省道二期建设。服务园区和农村发展，做好350省道前期工作，启动褒禅山香泉旅游度假区内部路网建设，实施县乡公路、乡村公路畅通工程，改造50公里县乡公路、150公里乡村公路。

****着力提升城镇功能品质。****认真落实“一尊重、五统筹”总体要求，坚持“组团式、网格化、田园城市”发展思路，全面推进城镇“三治三增三提升”行动，着力抓好主城区、三县县城和重点镇规划建设管理工作。强化“规划即法”意识，完成城市总体规划修改，实现控规全覆盖，完善各类专项规划，注重加强城市设计，着力塑造城市特色风貌，让城市生活更美好。加强城区路网建设，续建慈湖河路改造、冯桥路和江东大道南延工程，实施红旗路贯通、九华东路二期工程。推进综合客运中心枢纽站、体育会展中心等公共设施建设。精心精细管理城市，推动城市管理扁平化，重视解决城区部分道路拥堵和停车难问题。开展“多规合一”、海绵城市和城市地下综合管廊改造试点。新增和改造绿化面积600万平方米，建成街头游园15个、城市绿道55公里。改造棚户区1.1万户，改善群众居住环境。

****扎实推进美好乡村建设。****大力发展现代都市农业，引导农产品加工业集聚发展，推进农村一二三产融合发展，实现农产品加工产值增长10%。健全新型职业农民培育机制，创建农业产业化联合体，完善农业信贷担保机制，构建农业资源循环利用体系，打造姑溪河大闸蟹、和县蔬菜、含山大米等区域公共品牌。加快水阳江下游、裕溪河防洪治理等重点水利工程建设，力争长江马鞍山河段二期整治工程开工建设。推进16个乡镇政府驻地建成区和44个省级中心村建设，深入实施“三线三边”环境综合整治，大力整治农村环境，扎实做好城乡违法建设管控工作。实施乡村旅游提升工程，发展乡村休闲体验游，争创省级乡村旅游示范村5个，新增星级农家乐30家。

（五）持续建设绿色生态城市。坚持尊重自然、顺应自然，有度有序利用自然，加快建设资源节约型、环境友好型社会，打造绿色生态家园。

****加强生态文明建设。****深入推进生态市创建行动计划，坚持项目带动，推进重点企业节能减排、城镇污水垃圾处理等10个重点生态项目；夯实基层基础，支持当涂县争创省级生态文明建设示范县、和县建成省级生态县，创建一批省级和国家级生态乡镇、村。全面完成千万亩森林增长工程。优先发展公共交通，推进公共自行车二期项目建设，鼓励绿色低碳出行。积极推广建筑产业化和绿色建筑。加快生活垃圾焚烧发电项目建设，开工餐厨垃圾收运处理项目。支持市经开区创建国家生态工业示范园区，推进慈湖高新区资源综合利用和循环化改造项目。

****强化环境综合治理。****严格落实《环境保护法》和《安徽省环境保护条例》，加强环境执法，巩固突出环境问题专项整治成果，加快淘汰落后产能，为推动产业升级腾出空间。加强大气污染防治，推进空气质量预警体系建设，实施燃煤电厂超低排放和节能改造，基本淘汰黄标车，加强秸秆禁烧和综合利用，PM10平均浓度持续下降。采用PPP方式开展城区水环境综合治理，整治永丰河、东湖等4条水系，改造4座泵站，提升排涝标准，改善水质环境。推进慈湖河流域水环境治理利用世行贷款项目，修复慈湖河流域生态环境。建成污水处理配套管网50公里。开展土壤污染治理和修复试点。

****节约高效利用资源。****积极发展新能源产业，推进银阳光伏、茂迪太阳能电池及组件制造等项目建设。继续做好能源消费“双控”工作，支持当涂县申报大唐电厂二期项目。开展全民节能行动计划，加强重点领域和公共机构节能，推行合同能源管理。深化法治国土建设和“四模创建”活动，继续开展批而未供、供而未用土地清理处置工作，盘活存量土地，加强矿山管理，做好不动产统一登记工作。强化用水定额管理，完善阶梯水价制度，开展城市节水综合改造示范，提高水资源配置和利用效率。

（六）持续提高群众幸福指数。按照人人参与、人人尽力、人人享有的要求，坚守底线、突出重点、完善制度、引导预期，努力让发展成果更多更公平地惠及全市人民。

****着力保障改善民生。****在实施好省里民生工程基础上，重点办好公交服务升级、残疾人康复托养、淘汰黄标车及老旧车、城市水系综合治理、老旧小区提升改造、农村卫生室达标等6件民生实事，通过办好每一件具体实事，让老百姓得到更多的实惠。坚持精准扶贫、精准脱贫，确保完成省政府下达的脱贫任务。完善就业服务，支持灵活就业和新就业形态，全年新增就业岗位4.3万人。推进全民参保计划全国试点，确保社会保险参保率保持在96%以上。统筹各类社会救助资源，提升社会救助规范化水平，关爱留守妇女儿童，维护残疾人合法权益，做好帮扶困难职工和农民工工作，支持慈善公益事业发展。继续保持物价水平总体稳定。

****强化基本公共服务。****大力发展学前教育，鼓励普惠性幼儿园发展，通过集团化办学推动优质教育均衡发展，加强城乡教师队伍建设和互动交流，科学设置学校布局和办学规模。深化职业教育产教融合、工学结合、校企合作，培育更多适应我市产业发展需要的应用型人才。巩固城市和县级公立医院改革成果，推进分级诊疗体系建设。妇幼保健院新门诊楼交付使用，秀山医院基本建成。实施好全面两孩政策，完善计生服务管理。大力弘扬社会主义核心价值观，加强思想道德和社会诚信建设，广泛开展志愿服务，让我们这座城市处处充满文明之风。推进国家公共文化服务标准化试点，建成10个乡镇综合文化服务中心、100个村级文化服务中心，力争创作1-2个文艺精品冲刺省“五个一”工程奖。广泛开展全民健身活动。深入推进统计工作规范化，全力做好第三次全国农业普查。繁荣发展社会科学、文学艺术、新闻出版、广播影视，加强档案和地方志工作，促进防灾减灾、红十字、气象现代化等事业协调发展。

****提升社会治理能力。****实施“七五”普法规划，加强律师行业建设，完善法律服务体系，推进法治社会建设。深化和谐社区建设，增强社区自治和服务功能，推动社会治理重心下移。夯实信访工作基层基础，推进网上信访，化解信访积案，依法维护群众合法权益。完善立体化现代化社会治安防控体系，不断提升“平安马鞍山”建设水平。继续开展安全生产“铸安”行动，坚决遏制较大以上生产安全事故发生。认真落实粮食安全责任制。严格食品药品安全监管，让人民群众生活更健康、更安全。健全应急管理机制，提高突发事件应急处置能力。推动民族团结进步事业健康发展，维护宗教领域和谐稳定。深入开展国防教育，加强国防动员和双拥工作，提高军民融合发展水平。

（七）持续加强政府自身建设。以改革的精神、务实的态度、法治的方式、创新的方法、扎实的作风，积极推动政府职能转变，促进政府治理体系和治理能力现代化。

****深入推进改革。****围绕“放管服”要求，深化行政管理体制改革，严格落实“三个清单”制度，建立动态管理机制。启用市政务服务中心新办事大厅，持续推进行政审批权相对集中改革，推动“互联网+政务服务”，健全完善社会服务管理信息化平台。进一步深化商事登记制度改革，开展注册登记电子化。推进农村综合改革，完成农村土地确权登记颁证，稳妥推进农村集体资产股份制改革，深入推进供销社综合体制改革，拓展农产品交易市场，激发农业发展活力。建立全面规范、公开透明预算制度，盘活存量资金，提高财政资金绩效水平。完善全口径政府债务管理，做好存量债务置换，规范新增债务举借，防范和化解债务风险。完善江东控股集团管理体制和法人治理结构，打造国有资本投资控股集团，带动政府融资平台市场化转型。

****建设法治政府。****带头尊法学法守法用法，及时跟进政府立法工作，健全重大决策合法性审查机制，依法规范决策行为。深化行政执法体制改革，推进重点领域综合执法，全面落实行政执法责任制。发挥行政复议监督纠错功能，认真做好行政应诉工作。自觉接受市人大的法律监督、工作监督和市政协的民主监督，坚持定期向市人大常委会报告工作，向市政协通报情况，加强与各民主党派、工商联、无党派人士和人民团体对口联系，主动接受社会监督、舆论监督。深化政务公开，让权力在阳光下运行。

****提高行政效能。****始终把干事创业谋发展作为最大责任，夙夜在公、勤勉工作，敢于担当、勇于负责。既要脚踏实地、埋头苦干，又要仰望天空、着眼未来，抢抓发展先机，争取战略主动。狠抓效能建设，增强服务意识，紧盯目标干，围绕项目转，推动招商引资、服务企业、项目建设等重点工作取得实效。坚持问题导向，推行“一线工作法”，到基层一线去发现问题、研究问题、解决问题。狠抓工作落实，强化督促检查，确保每项工作善始善终、善做善成。密切联系群众，倾听群众呼声，回应群众关切，多做打基础、利长远的工作，多办顺民心、惠民生的实事，努力创造经得起实践、人民和历史检验的业绩。

****永葆清正廉洁。****认真抓好政府系统党建工作，巩固党的群众路线教育实践活动和“三严三实”专题教育成果。严格履行“一岗双责”，全面落实党风廉政建设责任制，加强行政监察，推进审计监督和制度规范全覆盖，强化对重点领域和关键环节的监督制约，用制度管权管事管人，严肃查处发生在群众身边的“四风”和腐败问题。厉行勤俭节约，反对铺张浪费，严控“三公经费”支出，把有限的财力用于发展经济、保障民生，着力打造为民务实清廉政府。

各位代表，新常态新形势催人奋进，新目标新征程任重道远。让我们在省委、省政府和市委的坚强领导下，团结拼搏，攻坚克难，干在实处，走在前列，让我们的城市一年比一年更美好，人民一年比一年更幸福，为全面建成更高水平小康社会、创造更加美好明天而努力奋斗！
